# Presentation of an online programme for children refugees: pilot study in Greece

**DOI:** 10.1080/20008198.2017.1351197

**Published:** 2017-09-29

**Authors:** Anastasia Kalantzi-Azizi, Theodora Anastasiou

**Affiliations:** ^a^ Institute of Behavioural Research and Therapy, Athens, Greece

**Keywords:** Refugee, trauma, children and adolescents, interventions

## Abstract

**Background**: The psychological trauma caused by war to children and adolescents is of great concern to specialists, especially in recent years. At the end of 2014, from almost 60 million forcibly displaced people all over the world, one third were refugees and half of them were minors (Turner, 2015). It is widely known that a great percentage of them suffer from mental problems (Vostanis, 2016). Post-traumatic stress disorder, depression and severe anxiety disorders are among the most common mental disorders that occur in refugee minors. Few relevant and empirically documented surveys exist though. With regard to interventions, mainly we take advantage of our experience after other traumatic events such as natural disasters, sexual abuse, etc.

**Objective**: There is a lack of preventive screening and psycho-educational interventions in Greece and that is why we adapted an online preventive manual ‘The child and the liberation from the shadow of the terrible big fear’, a self-help trauma picture book for parents and their children who fled from war and home (Stein et al., 2017), available in nine languages already (www.susannestein.de).

**Method**: We tested the feasibility of using this preventive manual in a small sample of children/adolescents (*N* = 6) of Afghan origin in the summer of 2016 (refugee camp of Agios Andreas, Nea Makri). This programme covers a gap in primary prevention but also prepares the child for psychotherapy, if needed.

This manual consists of four parts and one appendix:

A. An illustrated story about a child who, due to war, is exposed to a terrible anxiety situation and is forced to leave with his parents for a ‘safe’ country, but the shadow of the great fear follows. In the end, it is relieved of this shadow by various means of support/assistance (pp. 4–29).

B. A series of recommendations and psychoeducation for parents etc. (pp. 30–37). For example, ‘What this big fear can do to your child?’ or ‘What can make the child stronger?’

C. Relevant themes for painting (pp. 38–43). For example ‘What can you do really well?’, ‘Who are the people you love?’ and ‘Can you paint a difficult situation from your past that you remember?’

D. Information about the author and other relevant organizations (pp. 44–49).

Appendix: Support material for the use of the book by people without trauma training (pp. 50–52).

This material is addressing parents and children – 10–15 years old – who have experienced war and have been forced to leave their country of origin. Lately the material is also addressing those who are active in organizations and camps with refugee children (adult caregivers), but also specialists (psychologists, psychotherapists, psychiatrists, paediatricians, etc.) and teachers.

**Results**: None of the children dropped out and, from the qualitative evaluation, we conclude that the parents seem to be able to become more engaged, the children respond positively and also psychological difficulties can be identified early on (from the six children of this pilot research, only three need further psychological support).

**Conclusions**: It seems feasible to use this online preventive manual in Afghan refugees in Greece. Further research will need to determine its efficacy. Such programmes can only be effective in a safe environment where there will be no fear of repatriation and the basic needs are covered. The above experiences have led the researchers of this pilot study to create a trauma group at the Institute of Behavioural Research & Therapy in Greece in order to further address preventive interventions for refugees. The trauma team recently became a member of the NOW-Network (www.now-conference.org).
